# Molecular Dynamics Calculations of Grain Boundary Mobility in CdTe

**DOI:** 10.3390/nano9040552

**Published:** 2019-04-04

**Authors:** Rodolfo Aguirre, Sharmin Abdullah, Xiaowang Zhou, David Zubia

**Affiliations:** 1 Department of Electrical and Computer Engineering, University of Texas at El Paso, El Paso, TX 79968, USA; sabdullah@miners.utep.edu (S.A.); dzubia@utep.edu (D.Z.); 2 Mechanics of Materials Department, Sandia National Laboratories, Livermore, CA 94550, USA; xzhou@sandia.gov

**Keywords:** Molecular Dynamics, Grain Boundaries, CdTe solar cells, thin films

## Abstract

Molecular dynamics (MD) simulations have been applied to study mobilities of Σ3, Σ7 and Σ11 grain boundaries in CdTe. First, an existing MD approach to drive the motion of grain boundaries in face-centered-cubic and body-centered-cubic crystals was generalized for arbitrary crystals. MD simulations were next performed to calculate grain boundary velocities in CdTe crystals at different temperatures, driving forces, and grain boundary terminations. Here a grain boundary is said to be Te-terminated if its migration encounters sequentially Cd·Te−Cd·Te… planes, where “·” and “−” represent short and long spacing respectively. Likewise, a grain boundary is said to be Cd-terminated if its migration encounters sequentially Te·Cd−Te·Cd… planes. Grain boundary mobility laws, suitable for engineering time and length scales, were then obtained by fitting the MD results to Arrhenius equation. These studies indicated that the Σ3 grain boundary has significantly lower mobility than the Σ7 and Σ11 grain boundaries. The Σ7 Te-terminated grain boundary has lower mobility than the Σ7 Cd-terminated grain boundary, and that the Σ11 Cd-terminated grain boundary has lower mobility than the Σ11 Te-terminated grain boundary.

## 1. Introduction

Mobilities of grain boundaries are important properties in materials science and engineering because they determine grain structures under given processing and operating conditions. As an example, grain boundaries (GB) are known to sensitively affect the performance of CdTe/CdS solar cells. While there are an unlimited number of GB types and each type may have a different effect, studies have shown that Σ3 type or twin GBs in CdTe are usually not active in carrier recombination [1]. On the other hand, non-Σ3 type GBs in CdTe often act as non-radiative recombination centers [2]. However, grain structures in CdTe/CdS systems are complex, involving variations in the grain sizes and shapes, GB types (e.g., Σ value, GB plane, etc.), GB polar characteristics (e.g., Cd-termination or Te-termination), and defects (e.g., vacancies and distortions) along the GB. To optimize CdTe/CdS solar cell performance, predictive simulations of grain structure evolution is highly desired.

Recently, we demonstrated that molecular dynamics (MD) simulations of vapor deposition could mimic the formation of polycrystalline CdS films on amorphous CdS substrates without any prior assumptions [3]. Despite similar grain morphologies to experimental observations, the predicted grain sizes are much smaller [4] due to the short MD time and length scales. One way to address this issue is to use MD simulations to calculate GB velocities at different temperatures and driving forces, and then to derive a GB mobility law at engineering time and length scales by fitting the MD results to an Arrhenius equation. This mobility law can then be used in large scale models to predict grain structure evolution that is comparable to experiments. A significant advantage of MD simulations is that they can separately study each GB type whereas the GB mobilities measured in experiments usually represent average behavior of all GB types.

In order to calculate GB velocities, a driving force must be applied to cause a sufficient migration of the GB within the short MD time scale. In some previous studies, GB curvature has been used as a driving force [5,6]. Such an approach requires a large system size in order to get the size-independent results. In addition, curved grain boundaries do not distinguish different GB types that are always planar, such as Σ3{111}, Σ5{100}, etc. To solve this problem, Janssens et al. [7] developed an algorithm that raises the energy of one grain with respect to the other and demonstrated that this provides a sufficient driving force to cause the motion of GB during MD simulations. The approach proposed by Janssens et al., however, is only applicable to face-centered-cubic (FCC) and body-centered-cubic (BCC) crystals [7].

The objective of the present work is threefold: (a) generalize the approach by Janssens et al. [7] for arbitrary crystals; (b) perform MD simulations to derive GB mobility laws for Σ3, Σ7, and Σ11 grain boundaries in CdTe; and (c) mechanistically examine the GB migration to achieve better understanding of mobility of different GB types in CdTe. Σ3 GB was studied because it is the most widely observed GB [8]. Σ7 and Σ11 grain boundaries were chosen for study because they are also frequently observed but in lower abundance compared to Σ3 [2] and their slightly higher Σ values provide a nice contrast to the Σ3 GB.

## 2. Methods

### 2.1. Molecular Dynamics Simulation Algorithm for Grain Boundary Migration

The approach developed by Janssens et al. [7] for GB migration has been implemented as the “orient/fcc” and “orient/bcc” fix styles in the Large-scale Atomic/Molecular Massively Parallel Simulator (LAMMPS) MD code [9,10] for FCC and BCC crystals respectively. However, CdTe has a zinc-blende (ZB) crystal structure. To generalize the method for various crystals, we first briefly summarize the mathematical formulation of the method [7].

Consider a bi-crystal system composed of two grains with orientation *I* and *J* respectively. If atoms are on ideal lattice sites, each atom *i* in grain *I* has a list of neighbors that are displaced from atom *i* by vectors of Δ***r**^I^*_j_, and each atom *i* in grain *J* has a list of neighbors that are displaced from atom *i* by vectors of Δ***r**^J^*_j_, where *j = i_1_, i_2_, …, i_N_* represent the total number of neighbors of atom *i*. In the absence of defects and thermal vibrations, the orientation difference between grains *I* and *J* can be quantified by a deviation parameter:

(1) $$\xi_{IJ}=\sum_{j=i_{1}}^{i_{N}}\left|\Delta r_{j}^{J}-\Delta r_{j}^{I}\right|,$$

when the orientations of *I* and *J* are given, Δ***r**^I^_j_* and Δ***r**^J^_j_* are known, and therefore *ξ_IJ_* is a constant. During an MD simulation, each atom *i* (in either grain *I* or *J*) has a list of neighbors that are displaced from atom *i* by vectors of Δ***r**_j_, j = i_1_, i_2_, …, i_N_*. Without losing generality, we use orientation *I* as the reference. The deviation of atom *i* from the orientation *I* can then be quantified by an ordering parameter:

(2) $$\xi_{i}=\sum_{j=i_{1}}^{i_{N}}\left|\Delta r_{j}-\Delta r_{j}^{I}\right|,$$

*ξ_i_* varies during simulation as atoms move. To introduce a driving force for GB migration, an energy is applied to atoms in grain *J* but not in grain *I*. This is achieved by adding the following smooth potential function:

(3) $$u=\{\begin{array}{ll} 0, & \xi_{i}/\xi_{IJ}<f\\ \frac{V}{2}\left[1-\cos\left(\frac{\xi_{i}/\xi_{IJ}-f}{1-2f}\pi \right)\right], & \xi_{IJ}\leq \xi_{i}/\xi_{IJ}\leq 1-f\\ V, & \xi_{i}/\xi_{IJ}>1-f \end{array},$$

where *V* is a parameter representing the energy applied to each atom in grain *J*, and *f* is another parameter. If all atoms are at ideal positions, we can assume that *f* = 0. If this is the case, atom *i* is in grain *I* whenever *ξ_i_/ξ_IJ_* = *f* (i.e., *ξ_i_* = 0) and in grain *J* whenever *ξ_i_/ξ_IJ_* = 1 − *f* (i.e., *ξ_i_ = ξ_IJ_*) as stated above. Practically, atoms are never at ideal positions due to thermal vibrations. Hence, f is taken as a positive buffer number (e.g., *f* = 0.25) so that atom *i* is in grain *I* when *ξ_i_/ξ_IJ_* < *f* (i.e., *ξ_i_* is close to zero) and in grain *J* when *ξ_i_/ξ_IJ_* > 1 − *f* (i.e., *ξ_i_* is close to *ξ_IJ_*). It can be seen from Equation (3) that when atoms are in grain *I, ξ_i_/ξ_IJ_* < f, no additional energy is applied; when atoms are in grain *J, ξ_i_/ξ*_IJ_ > 1 − *f*, a constant energy *V* is added to each atom; and when atoms are near the grain boundary, *f* ≤ *ξ_i_/ξ_IJ_* ≤ 1 − *f*, the added energy smoothly changes from 0 at the side of grain *I* to *V* at the side of grain *J*.

In the LAMMPS “orient/fcc” and “orient/bcc” fix styles, the algorithm described above is implemented using a fixed neighbor list *i_1_, i_2_, …, i_N_* that is taken from FCC and BCC crystals respectively. We have implemented a new LAMMPS fix style “orient/general” to enable users to define the neighbor list. As a result, our fix style can be applied to arbitrary crystals. Note that the nearest neighbor vectors Δ***r**^I^*_j_ cannot be uniquely defined for non-centrosymmetric crystals such as zinc-blende crystals. This can be easily addressed if the neighbor list *j = i_1_, i_2_, …, i_N_* that users define includes only the second nearest neighbors.

### 2.2. Interatomic Potential

A Stillinger-Weber (SW) potential [11,12] was used in our simulations. This potential is chosen because it both captures accurately the experimental atomic volumes, cohesive energies, and elastic properties of CdTe [12], and predicts the crystalline growth of II-VI compounds correctly [13]. Since the potential has been widely used to study grain structures in CdTe/CdS systems [3,13,14,15,16], knowledge of GB mobilities using the same potential has an additional advantage for comparing to previous studies.

### 2.3. Molecular Dynamics Conditions

**Figure 1:**
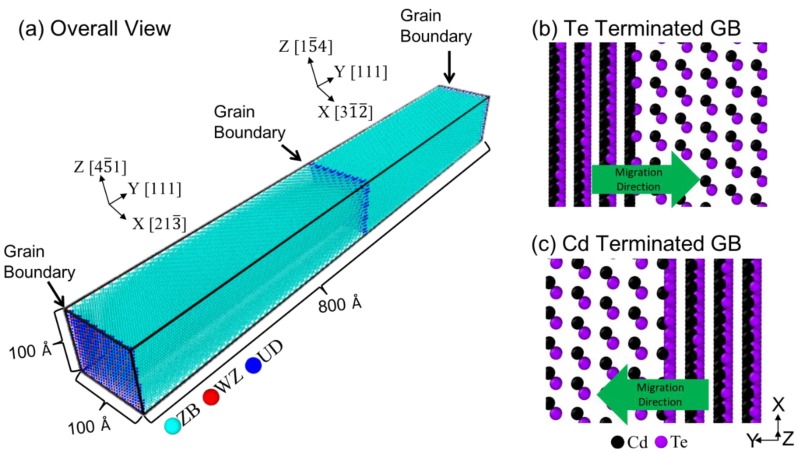
Computational cell for a Σ7 grain boundary in CdTe: (**a**) a 3D view; (**b**) the gain boundary at the two ends of the periodic cell in (**a**) showing Te-termination as the migration of the boundary encounters the Cd·Te−Cd·Te… plane sequence; and (**c**) the grain boundary in the center of the cell in (**a**) showing the Cd-termination where the migration of the boundary encounters the Te·Cd−Te·Cd plane sequence.

Our MD simulations were performed using LAMMPS on the Comet computer cluster at the San Diego Supercomputer Center (SDSC) under the Extreme Science and Engineering Discovery Environment (XSEDE) program [17]. Three CdTe grain boundaries Σ3{111}, Σ7{111}, and Σ11{311} were considered. For simplicity, the grain boundaries will be referenced as Σ3, Σ7, and Σ11 in the following without including the plane indices. Using the Σ7 GB as an example, the computational systems are illustrated in Figure 1a, where light blue, red, and dark blue indicate atoms within zinc-blende (ZB), wurtzite (WZ), and undetermined (UD) lattice sites, respectively. Under the periodic boundary conditions, the simulation boxes contained two CdTe grains with two infinite grain boundaries parallel to the X-Z plane, one at the center, and the other one at the end. These two boundaries, however, are not necessarily equivalent. To examine this, the blowout view of the two Σ7 GBs are shown respectively in Figure 1b,c, where the green arrows indicate the GB migration directions. It can be seen that the GB shown in Figure 1b is terminated by Te atoms and its migration encounters Cd·Te−Cd·Te… plane sequence, where “·” and “–” represent short and long plane spacings. In contrast, the GB shown in Figure 1c is terminated by Cd atoms and its migration encounters Te·Cd−Te·Cd … plane sequence. This occurs because the Σ7 GB is polar. We will study Te-terminated and Cd-terminated GBs separately because there is no reason to assume that the two types of GBs have the same mobility.

All computational boxes used ~100 × 100 Å^2^ dimensions on X-Z planes and ~800 Å in the Y direction. These structures were constructed using the “createAtoms” tool in the LAMMPS package. Using isothermal-isobaric (NPT) ensemble, MD simulations were performed at various temperatures T and energies V for a total of 0.2 ns, and GB velocities were calculated by tracking displacement of grain boundaries as a function of time.

## 3. Molecular Dynamics Results

The key to successful MD simulations is to select an appropriate set of temperatures T and energies V that results in thermally activated migration of grain boundaries. Because thermally activated migration can occur at different conditions for different grain boundaries, trial runs were performed to identify appropriate temperatures and energies for simulations. Based on the trial runs, MD studies of the Σ3, Σ7, and Σ11 grain boundaries were performed at a matrix of five temperatures (1600, 1700, 1800, 1900, and 2000 K) and three energies (0.75, 1.00, and 1.25 eV). Note that because the interatomic potential we used significantly overestimates the experimental melting point of CdTe, comparison with experiments should be made using homologous temperatures. Since we are interested in trends, we did not make this conversion for clarity.

**Figure 2:**
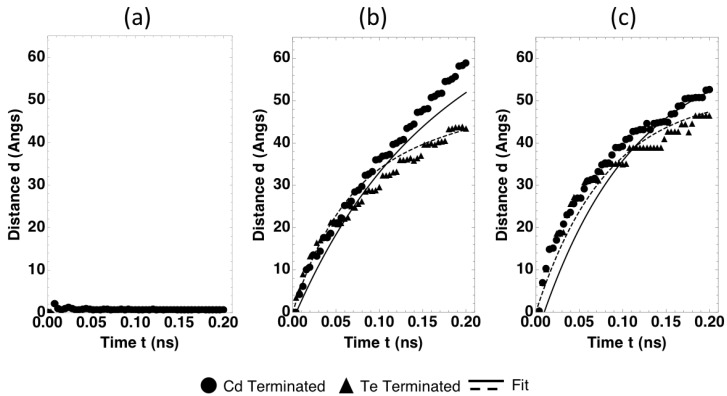
GB migration distance as a function of time for (**a**) Σ3, (**b**) Σ7, and (**c**) Σ11 grain boundaries at temperature and energy of 2000 K and 1 eV. Black circles and triangles show the MD results for the Cd and Te terminated GBs, respectively, with the solid and dash lines representing the corresponding predictions from the fitted functions.

Post-processing analysis of MD results was performed to obtain GB migration distance as a function of time. Using a temperature of T = 2000 K and energy of V = 1.0 eV as the example cases, the final results of GB migration distance as a function of time are shown as the data points in Figure 2a–c for the Σ3, Σ7, and Σ11 grain boundaries, respectively. Here the black circles and triangles represent the MD data of the Cd terminated, and Te terminated GBs respectively, and the solid and dash lines are corresponding Arrhenius fits to be described below.

Figure 2 reveals that the migration distance of the Σ3 grain boundary remains small within the 0.2 ns simulated time. This suggests that the migration energy barrier of the Σ3 grain boundary is high, which results in low mobility. On the other hand, the migration distances of the Σ7 and Σ11 grain boundaries are significant, suggesting that the migration energy barriers of these two grain boundaries are relatively low. Further examination of our MD data obtained at other temperatures and energies (our trial MD simulations cover temperatures from 300 K to 2200 K and energies up to 2 eV) indicated that within the short-time MD simulations, either the migration distance of the Σ3 grain boundary remains small or the system becomes melted. On the other hand, within the temperature and energy ranges reported here, the migration distances of the Σ7 and Σ11 grains boundaries increase when temperature and driving energy are increased. This validates that our MD simulation conditions capture the thermally activated migration for these two grains boundaries. We will derive mobility laws for these two GBs in the next section.

## 4. Arrhenius Mobility Law

The MD results presented above indicate that the Σ3 GB has much lower mobility than other higher Σ GBs. This means that Σ3 GBs are more likely to be retained during annealing, which is consistent with the experimental work by Moutinho et al. where CdTe samples showed 71% of the GBs are Σ3 [18]. The lack of Σ3 GB migration in simulations does not mean that the boundary will never move during experiments. It just means that the short-time MD simulations did not provide sufficient information to derive a mobility law for the Σ3 GB. Without a specifically derived mobility law for the Σ3 GB, microstructural evolution of CdTe crystals can be approximately modelled by assuming much lower mobility for the Σ3 grain boundary as compared to other mobile GBs. Hence, we will derive the mobility laws only for the Σ7 and Σ11 grain boundaries. Notice that GB mobility in this work is referred to as the GB velocity *v* under a given set of temperature *T* and energy *V*. GB velocity can be directly related to the activation energy barrier *E_a_* by the Arrhenius formula:

(4) $$v=Ae^{-\;\frac{\left(E_{a}-V*c\right)}{kT}},$$

where *A* is a pre-exponential factor, *k* is the Boltzmann constant, and *c* is a constant. Due to the short time scale employed in the MD simulations, the GB migration distances (*d*) shown in Figure 2 are not linear with time (*t*). These non-linear distances can be well captured by the following equation:

(5) $$d=a_{0}\left(1-e^{-bt}\right)+at$$

where *a*_0_ is the excess/additional displacement due to higher non-equilibrated velocity, *b* is equal to 1/τ and represents the equilibration time constant, and *a* is the steady-state velocity. For an infinitely long system, GB remains invariant during migration. Hence, the system is expected to reach a steady state where the GB velocity approaches a constant. Indeed, when the time approaches *t* → ∞, Equation (5) describes a steady state linear migration correctly as: $d=a_{0}+at$, i.e., migration velocity is constant. Equation (5) introduces an additional term $e^{-bt}$ to capture the transition of a non-equilibrated MD starting state (e.g., the atom velocities are generated rather than simulated) to the steady state.

In order to fit Equation (4) based on Equation (5), we first derive a time-dependent GB velocity by differentiating Equation (5) with respect to *t*:

(6) $$v\left(t\right)=a_{0}be^{-bt}+a.$$

Equating Equations (4) and (6), we can solve *a*_0_ at *t* = 0. Substituting this *a*_0_ back in Equation (5), we arrive at:

(7) $$d=at-\frac{a}{b}+\frac{Ae^{-\frac{\left(E_{a}-cV\right)}{kT}}}{b}-\frac{Ae^{-\frac{\left(E_{a}-cV\right)}{kT}\mp bt}}{b}+\frac{ae^{-bt}}{b}.$$

Equation (7) describes GB migration distance as a function of time at different temperatures and energies. Fitting Equation (7) to all MD data allows us to determine all unknown parameters *E_a_*, *A*, *a*, *b*, *c* as shown in Table 1. The curves shown in Figure 2 are generated with the fitted parameters. In view that only five parameters are used to fit all MD data at different temperatures and energies and that the MD data involves statistical noises due to non-equilibration, the fitted functions represent the MD data fairly well. In fact, the fits were obtained from the ensemble of data at different temperature and energies.

Table 1 indicates that the Σ7 Te-terminated GB has a higher activation energy barrier than the Σ7 Cd-terminated GB. Correspondingly, the Σ7 Cd-terminated GB is seen to travel longer distances than Te-terminated GBs in our MD simulations. On the other hand, no correlation is found between the Σ value and GB mobility. For instance, the Σ7 GB has a higher barrier than the Σ11 GB in the Te-terminated cases, but the opposite is found for the Cd-terminated cases.

## 5. Discussion

### 5.1. Mechanistic Insights

**Figure 3:**
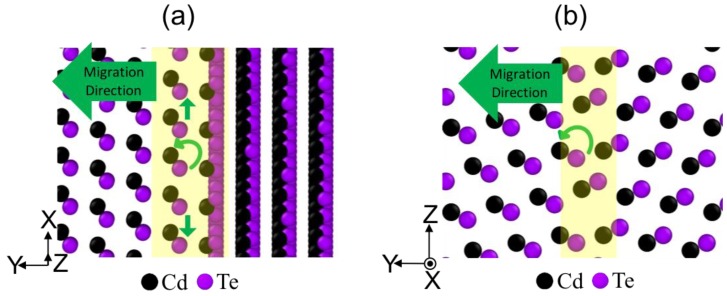
Motion of atoms during migration of (**a**) Σ7, and (**b**) Σ11 grain boundaries.

Time resolved MD simulations allow us to explore detailed migration mechanisms of various GBs. Figure 3a,b compares Σ7 and Σ11 GBs, where the yellow shaded regions highlight the boundary between the two grains and the small green arrows indicate the directions of atomic displacement observed from the MD simulations. For the Σ7 case shown in Figure 3a, two main GB migration mechanisms were observed. The first one involves a rotational motion between CdTe atomic pairs as indicated by the curved arrows, and the second one involves redistribution of Cd atoms between atomic layers as described by the straight arrows. In most cases, both mechanisms occurred concurrently, and only in a few cases, the rotational mechanism was observed first. Also, we observed that the GB migration started locally and propagated along the X-Z plane in the form of kinks. Once one atomic layer was fully reconstructed, the next atomic layer motion began.

For the Σ11 case shown in Figure 3b, the grain boundary migration occurs mainly by the rotational motion between CdTe atomic pairs with a few exceptions where redistribution of both Cd and Te atoms was observed. Formation of kinks is not observed as the propagation throughout the X-Z plane occurs almost simultaneously. Similar to the Σ7 GB, once one atomic layer was fully reconstructed, the next atomic layer motion began.

### 5.2. Body and Interfacial Driving Forces

In the present work, the grain boundary migration is driven by the body energy added to one grain with respect to the other. This is similar to the elastic energy stored in polycrystalline samples after deformation, which drives recrystallization and subsequent grain growth. In most grain growth processes, however, the grain boundary migration is driven by grain boundary energy itself in a direction to reduce the total grain boundary area. The grain boundary mobility due to boundary and body energies are not necessarily exactly the same, but they should correlate especially in terms of trends for different grain boundary types. To understand how our GB mobility law, derived under body energies, can be applied in cases under boundary energies, we discuss the correlation between the driving forces of boundary and body energies.

Consider a spherical grain with a radius of *r* and a grain boundary energy *σ* (in per area unit). The total boundary energy is *E = 4πr^2^σ*. The total force acting along the radius direction is *−∂E/∂r* = *−8πrσ*. The driving force in per area unit is therefore *f_σ_* = −*2σ/r*. On the other hand, consider planar grain with a cross-section area of *A*, a thickness of *t*, a density of *ρ* (atoms per volume), and body energy of *V* (energy per atom). The total body energy of this grain is *E = AtρV*. The total force acting along the thickness direction is *−∂E/∂t = −AρV*. The driving force in per area unit is, therefore, *f_V_ = −ρV*. Setting *f_σ_ = f_V_*, we can get a rough idea as to how our parameter *V* is related to grain boundary energy *σ* and grain size *r* in the spherical grain case.

## 6. Conclusions

This work developed a molecular dynamics simulation approach to study mobility of grain boundaries in arbitrary crystals and made the approach available in MD code LAMMPS. Extensive MD simulations were performed to study mobilities of Σ3, Σ7, and Σ11 grain boundaries in CdTe. We found that the high symmetry Σ3 GB is much less mobile than low symmetry Σ7 and Σ11 GBs. As a result, microstructures of CdTe crystals observed in experiments are likely to be composed of mainly Σ3 GBs whereas other low symmetry GBs tend to be annealed out. Based on the MD results, Arrhenius mobility laws were derived for the Σ7 and Σ11 GBs. These laws capture well the MD data, thereby permitting high-fidelity continuum or kinetic Monte Carlo simulations of microstructure evolution of CdTe crystals at engineering time and length scales. Our work also indicates that the Cd-terminated GB moves faster than the Te-terminated GB for the Σ7, but the opposite occurs for the Σ11 GB.

## Figures and Tables

**Table 1:** Fitted mobility parameters for the Σ7 and Σ11 GBs. The activation energy barrier of Σ3-111 would have been ∞, but this ∞ barrier is an artifact due to the lack of GB migration during the short MD time scale.

	Σ7-111 (Te Terminated)	Σ7-111 (Cd Terminated)	Σ11-311 (Te Terminated)	Σ11-311 (Cd Terminated)
***A* (m/s)**	5421.39	889.22	1342.19	3025.78
***a* (m/s)**	34.8394	44.7631	0.0001	0.0001
***b* (sec^−1^)**	16.519	7.52065	12.3306	9.34461
***c* (unitless)**	0.1825	0.175291	0.261754	0.240976
***E_a_* (eV)**	0.5	0.243296	0.324286	0.463243
